# P66. Generating and characterising WT1-specific T cells – research towards adoptive tumour therapy

**DOI:** 10.1186/2051-1426-2-S2-P40

**Published:** 2014-03-12

**Authors:** S Schmied, A Richter, M Assenmacher

**Affiliations:** 1 Miltenyi Biotec GmbH, Bergisch Gladbach, Germany

## Background

The Wilms tumour antigen 1 (WT1) is a self-antigen expressed at high levels in leukaemic cells, but not in healthy tissue. WT1, therefore, is a favourable target antigen for allogeneic T cell therapy to prevent leukaemic relapse after stem cell transplantation. However, a comprehensive characterisation of CD4^+^ and CD8^+^ WT1-specific T cells is missing and the efficient expansion of a polyclonal WT1-reactive T cell population for clinical use has remained a major challenge.

In this study we aim to directly *ex vivo* characterize WT1-specific T cells present in the blood of healthy donors at high-resolution and to develop a method for the rapid generation of functionally potent, polyclonal CD4^+^ and CD8^+^ WT1-specific T cells for clinical use.

## Methods

We utilise the magnetic enrichment of activation marker expressing cells after antigen-specific stimulation, as low frequencies of WT1-specific T cells in healthy donors do not allow direct detection.

## Results

*Ex vivo* frequencies of WT1-specific T cells range between 10^-6^ and 10^-5^ WT1-specific T cells within CD4^+^ T cells. In about 80% of healthy donors (n=15) a CD4^+^ memory response, accompanied by production of effector cytokines like IFN-γ and TNF-α against WT1 peptides is present. In contrast, detected CD137^+^CD8^+^ WT1-reactive T cells exhibit a naïve phenotype (CD45RA^+^CCR7^+^) in all tested donors (n=5).

An improved short-term expansion protocol to generate potent WT1-specific T cell cultures for clinical use was established utilising a CD137^+^ cell enrichment step. Notably, a high frequency of expanded CD4^+^ and CD8^+^ T cells show specific reactivity against WT1-presenting autologous cells as detected by production of effector cytokines after antigen-specific restimulation. Cytotoxic activity against antigen-loaded target cells could be shown by direct flow-cytometry-based cytotoxicity assays and antigen-specific upregulation of the degranulation marker CD107a. WT1-MHCI-Tetramerstainings furthermore confirmed antigen-specificity and suggested polyclonality within the CD8^+^ T cell population. In contrast to previous expansion protocols our polyclonally expanded T cells exhibit a favourable, unexhausted memory phenotype, express co-stimulatory markers CD27 and CD28 and the IL7R-a chain (CD127).

## Conclusions

Functional, polyclonal CD4^+^ and CD8^+^ WT1- reactive T cells can be efficiently enriched directly *ex vivo* from the natural repertoire by magnetic separation of T cells after antigen-specific stimulation. Thus, our approach holds great potential for the GMP-compliant generation of WT1-specific T cells for future clinical use.

